# Posterior Mitral Valve Hypoplasia: Three Clinical Cases and a Review of the Literature

**DOI:** 10.3390/biomedicines13051078

**Published:** 2025-04-29

**Authors:** Baudouin Koenig, Alin Ionescu, Elena Galli

**Affiliations:** Pôle d’Activité Médico-Chirurgicale Cardio-Vasculaire, Nouvel Hôpital Civil, Centre Hospitalier Universitaire, Université de Strasbourg, 67000 Strasbourg, France

**Keywords:** mitral valve hypoplasia, mitral regurgitation

## Abstract

**Background:** Posterior mitral leaflet hypoplasia is a rare condition, generally diagnosed in children. This paper presents three adult cases of posterior leaflet hypoplasia and comprehensively reviews the existing literature. **Methods and Results:** All patients presented with severe mitral regurgitation necessitating hospitalization. Transthoracic and transesophageal echocardiography were performed to determine the underlying etiology, revealing pronounced posterior mitral leaflet hypoplasia. Each case was evaluated by a multidisciplinary heart team. Surgical mitral valve intervention was feasible in two patients. In one high-risk patient, percutaneous treatment options for MR were explored but ultimately deemed unsuitable due to the anatomical characteristics of the valve. The patient was consequently managed conservatively with medical therapy. **Conclusions:** These cases demonstrate that posterior mitral leaflet hypoplasia may constitute an underrecognized cause of severe primary mitral regurgitation in adults. The management of such cases can be particularly challenging due to the atypical valvular morphology.

## 1. Introduction

Mitral regurgitation (MR) is the second most frequent valvular heart disease (VHD) in European countries after aortic stenosis [1,2]. MR generally occurs in adulthood and is classified as primary when resulting from degenerative or structural abnormalities of the mitral valve, and as secondary when caused by global or regional left ventricular remodeling and/or severe left atrial dilatation. Degenerative mitral valve disease, such as fibroelastic or Barlow’s disease, is the main cause of primary MR.

Less common etiologies of primary MR are infective endocarditis and rheumatic valve disease. Alongside these relatively well-known causes, it is important to acknowledge the existence of congenital mitral valve lesions [1,2].

Posterior mitral leaflet (PML) hypoplasia isreferred to as a rare congenital heart disease, usually detected in infants and childhood, with concomitant significant MR [3]. Until now, only 68 clinical cases have been described worldwide [3,4,5,6,7,8,9,10,11,12,13,14,15,16,17,18,19,20,21].

In this paper, we report a case series of three adult patients with severe primary MR due to PML hypoplasia, and we provide a review of the current literature on this uncommon condition.

## 2. Case Reports

### 2.1. Clinical Case 1

**Figure 1:**
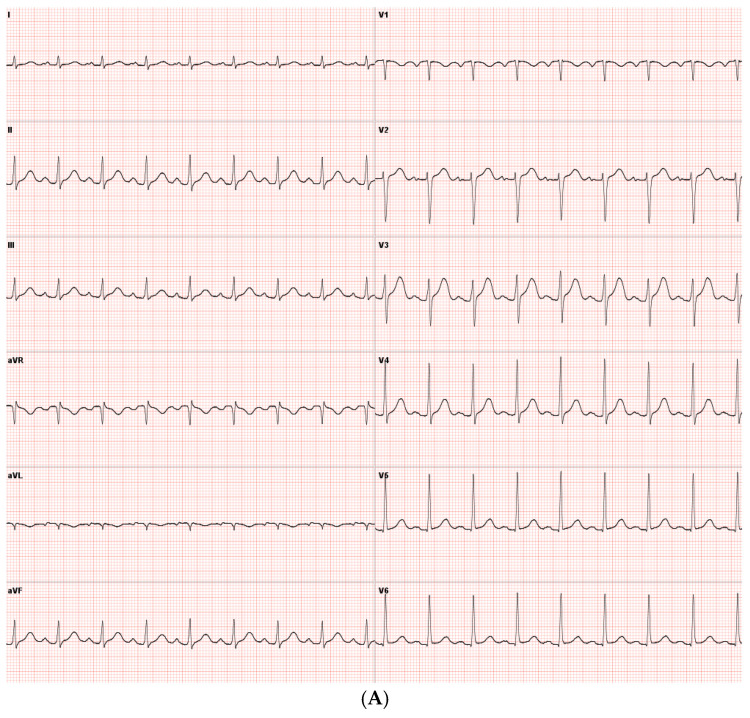

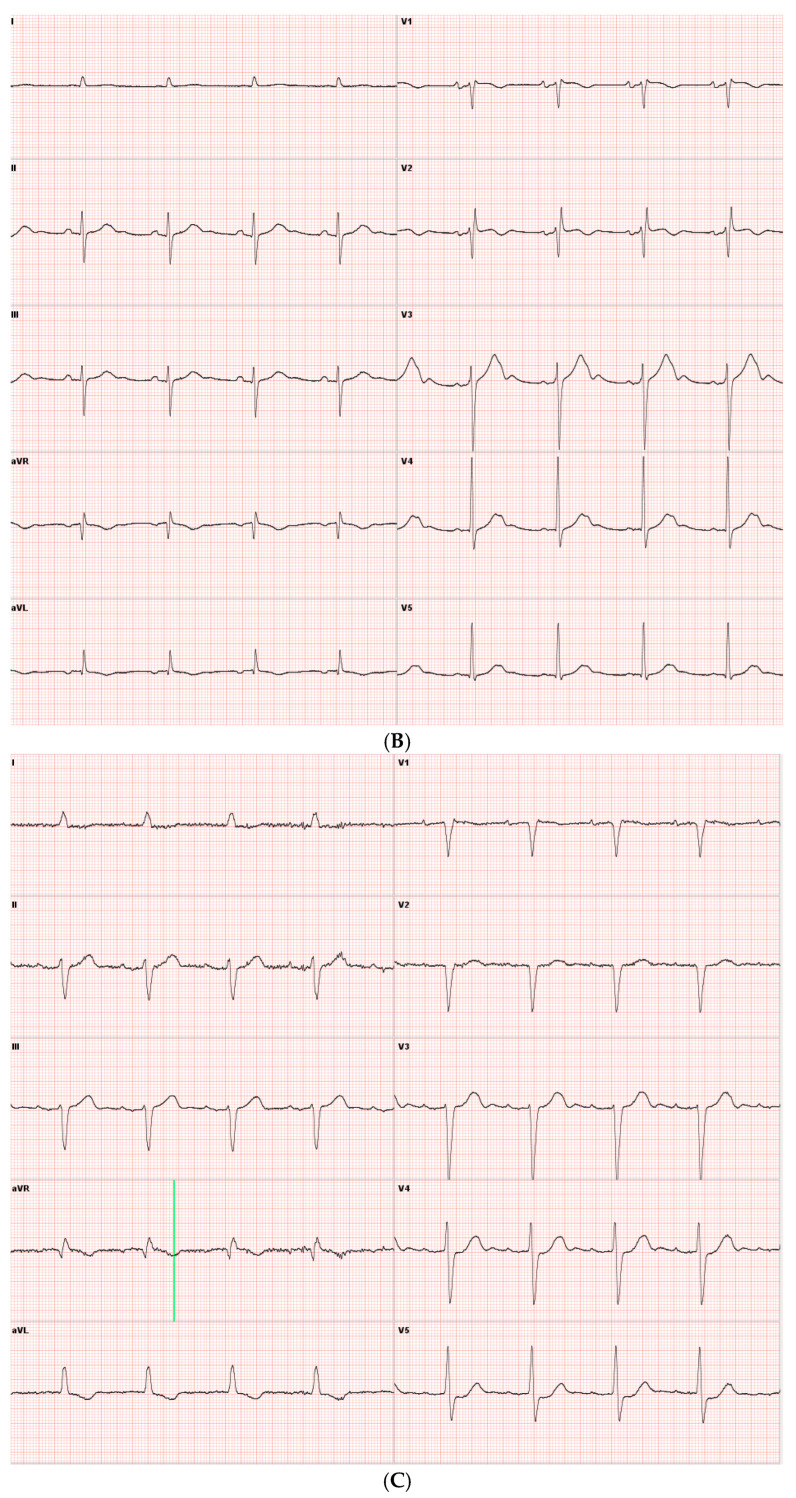
(**A**) ECG obtained in Case 1, showing sinus rhythm without signs of ischemia and normal left ventricular repolarization. (**B**) ECG obtained in Case 2 after atrial fibrillation cardioversion, showing sinus rhythm, incomplete right bundle branch block, no signs of ischemia, and normal left ventricular repolarization. (**C**) ECG obtained in Case 3, showing showed sinus rhythm and incomplete left bundle branch block.

**Figure 2:**
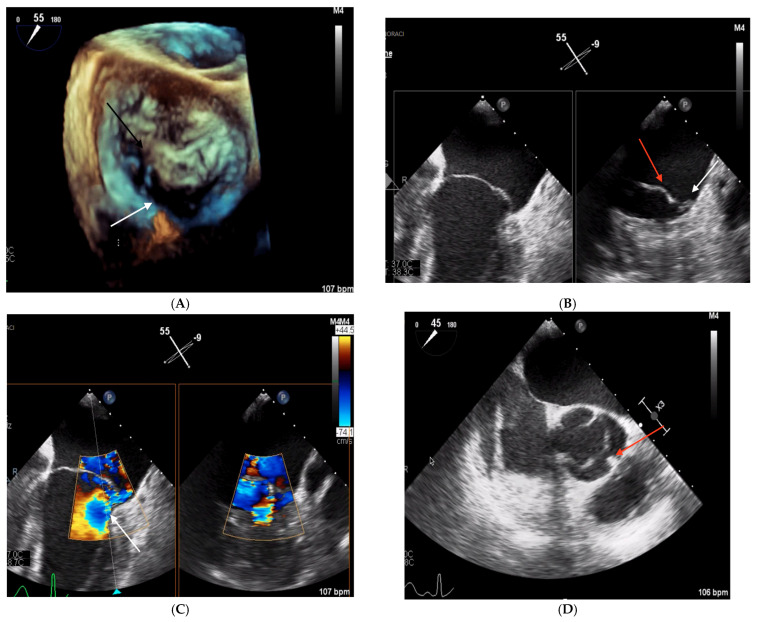
TEE images obtained in Clinical Case 1. (**A**) 3D TEE “en face” view of mitral valve showing a severely hypoplastic PML (white arrow) and an elongated and prolapsing AML (black arrow). (**B**) 2D TEE image at 55° mid-esophageal level (left panel) showing the elongated AML. The X plan in A1P1 (right panel) shows a large AML (red arrow) and severe PML hypoplasia (white arrow). (**C**) Color Doppler 2D TEE 55° mid-esophageal view (left panel) showing the severe, eccentric MR jet (white arrow) and the orthogonal plane crossing A1/P1, where the origin of the MR jet is located (right panel). (**D**) 2D TEE image at 45° showing type 1 L-R bicuspid aortic valve. The red arrow indicates cup fusion.

**Figure 3:**
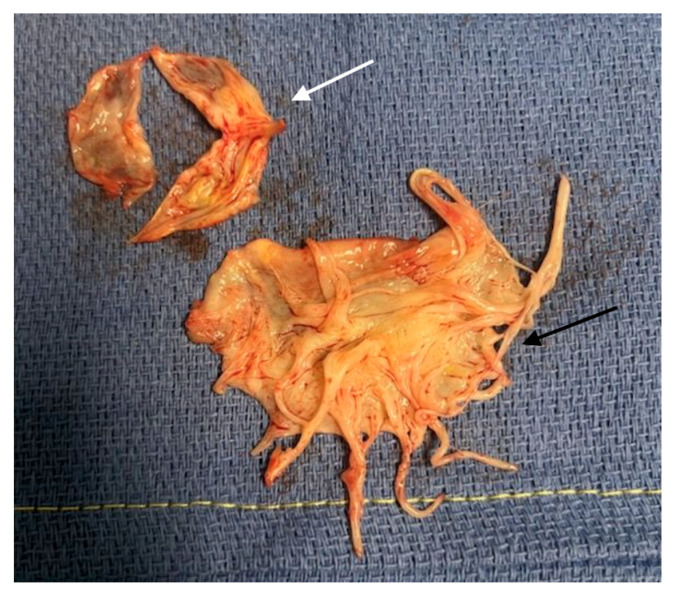
Operative samples of Case 1. The white arrow indicates the aortic valve sample showing the Type 1 L-R bicuspid aortic valve. The black arrow indicates the mixoïd AML sample.

A 44-year-old Caucasian man was admitted to the Intensive Care Unit (ICU) due to acute respiratory distress syndrome associated with pneumonia, requiring invasive mechanical ventilation. The electrocardiogram (ECG) at admission showed sinus tachycardia, without evidence of prior or ongoing ischemia and with normal left ventricular repolarization (Figure 1A). On physical examination, a 4/6 apical pansystolic murmur was detected, along with clinical signs of heart failure. The patient exhibited signs of an inflammatory syndrome, although blood cultures were negative. To rule out infective endocarditis, transthoracic (TTE) and transesophageal echocardiography (TEE) were performed. Although no vegetations were observed, imaging revealed significant mitral and aortic valve disease. Specifically, there was severe PML hypoplasia, while the anterior mitral leaflet (AML) was elongated, thickened, and prolapsing, resulting in severe primary mitral regurgitation (MR). Additionally, the patient was found to have a type I L-R bicuspid aortic valve with moderate aortic regurgitation (vena contracta 4 mm) (Figure 2, Table 1). Cardiac computed tomography confirmed the presence of a bicuspid aortic valve and excluded proximal aortic dilatation and coronary artery disease. A few months later, the patient underwent cardiac surgery. Mitral valve repair was not feasible due to the abnormal morphology of the PML, and the patient underwent concomitant mitral and aortic valve replacement with mechanical prostheses. The AML was myxomatous and exhibited multiple chordal ruptures, while the PML appeared small, retracted, and fibrotic. Operative specimens from the mitral and aortic valves are shown in Figure 3. The postoperative course was uneventful, and the patient was discharged seven days after surgery.

### 2.2. Clinical Case 2

A 76-year-old Caucasian woman was admitted to the emergency unit due to worsening heart failure and new-onset atrial fibrillation. Her medical history consisted of supraventricular arrhythmias and mild mitral regurgitation secondary to a mitral valve prolapse.

The ECG obtained after electrical cardioversion of atrial fibrillation showed incomplete right bundle branch withoutleft ventricular repolarization abnormalities (Figure 1B).

**Figure 4:**
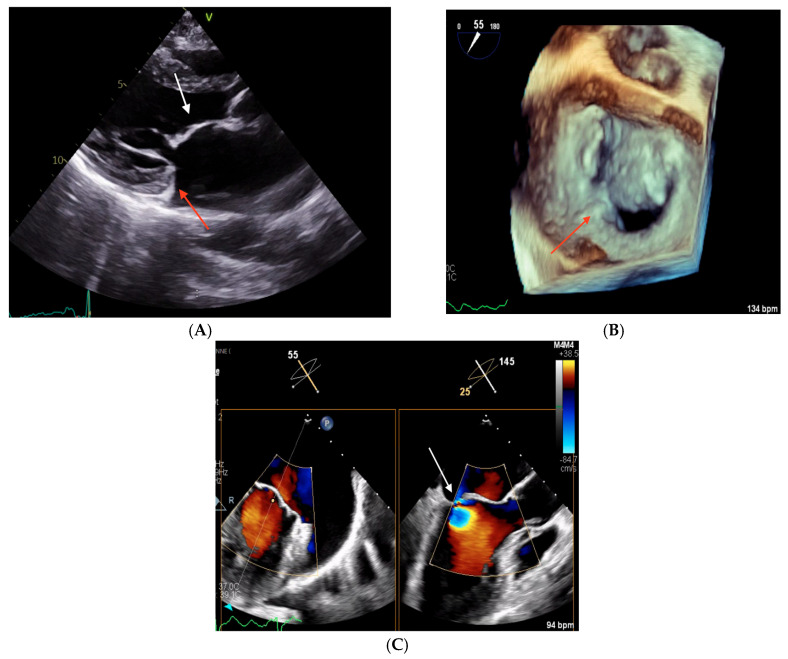
Echocardiographic images obtained in Clinical Case 2. (**A**) TTE parasternal long-axis view showing a thickened AML (white arrow) and an hypoplastic PML (red arrow). (**B**) 3D TEE “en face” view of the mitral valve showing severe PML hypoplasia with only a small P1 scallop (red arrow). (**C**) 2D TEE 55° mid-esophageal view (left panel) and the orthogonal plane crossing A2/P2, where the origin of the MR jet is located (right panel, white arrow).

On physical examination, a 4/6 systolic murmur was detected and the patient presented bilateral ankle swelling. TTE revealed a non-dilated left ventricle with normal kinetics and preserved left ventricular ejection fraction. The left atrium was severely dilated and there was at least moderate-to-severe primary mitral regurgitation (Table 1). TEE showed a thickened and elongated AML and a severely hypoplastic, rudimentary PML causing severe mitral regurgitation, without evidence of chordal rupture (Figure 4)

Coronary angiography ruled out significant coronary artery disease. The patient remained symptomatic despite diuretic therapy and atrial fibrillation cardioversion. Following a discussion between the heart team, trans-catheter edge-to-edge repair (TEER) of the mitral valve was judged unfeasible due to the abnormal morphology of the PML, and the patient underwent mitral valve replacement with a bioprosthesis.

### 2.3. Clinical Case 3

An 82-year-old Caucasian man underwent TTE because of increasing dyspnea and fatigue. The patient’s medical history included transcatheter aortic valve replacement (TAVR) for severe aortic stenosis and persistent atrial fibrillation. Physical examinations showed bilateral ankle edema and a 3/6 mitral systolic murmur.

The ECG showed sinus rhythm and incomplete left bundle branch block, without signs of previous ischemia (Figure 1C).

**Figure 5:**
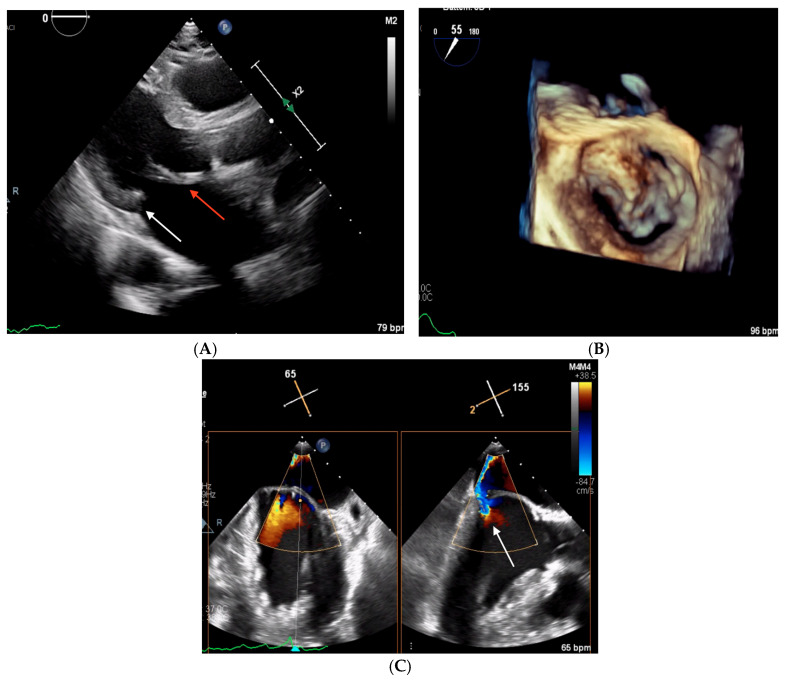
Echocardiographic images obtained in Clinical Case 3. (**A**) TTE parasternal long-axis view showing a thickened and elongated AML (red arrow) and the hypoplastic PML (white arrow). (**B**) 3D TEE “en face” view of mitral valve showing a severely hypoplastic PML and an AML prolapsing in A1 and A3. (**C**) Color Doppler 2D TEE 65° mid-esophageal view (left panel) and the orthogonal plane crossing A2/P2, where the origin of the MR jet is located (right panel, white arrow).

TTE showed a functional TAVR, a mildly dilated left ventricle, preservedleft ventricular ejection fraction, severely dilated left atrium, and a large eccentric MR jet (Table 1). TEE revealed PML hypoplasia and a large myxoid AML, and it confirmed the presence of severe eccentric primary MR (Figure 5). The surgical risk of the patient was high due to his advanced age and comorbidities (chronic kidney disease and respiratory insufficiency), discouraging conventional surgery.

The anatomy of the PML was deemed unsuitable for TEER. Transcatheter mitral valve implantation (TMVI) with the Tendyne^TM^ (Abbott Vascular, CA, USA) or HighLife^®^ (HighLife SAS, Paris, France) systems was not possible following screening failure. The patient was discharged under optimized medical therapy, including diuretics and dapagliflozin.

## 3. Discussion

PML hypoplasia is a complex entity, transitioning from a small-sized PML to complete PML agenesis, often referred to as a unileaflet mitral valve [22].

This mitral valve abnormality has been historically described in children and infants with symptomatic severe MR and is considered a cause of intrauterine death [23].

In a prospective analysis of 26.484 echocardiographic exams, Bar et al. found three cases of asymptomatic hypoplastic PML in young adults, corresponding to an overall prevalence of 1:8800 in asymptomatic patients [3].

After conducting a systematic literature search on PubMed, Scopus, Web of Science, and Google for case reports and articles on PML hypoplasia/aplasia, we found 68 cases of PML hypoplasia occurring in adults [4,5,6,7,8,9,10,11,12,13,14,15,16,17,18,19,20,21].

Patients’ clinical presentation was heterogeneous, going from asymptomatic disease (34 cases, 50%) to severe symptoms, including pulmonary edema at presentation (Table 2). PML hypoplasia was associated with concomitant congenital heart disease in 27 cases, including *ostrium secundum* atrial septal defect (ASD) [8 patients (12%)], *ostrium primum* ASD [1 patient (1%)], the presentation of a bicuspid aortic valve [16 patients (23%), with 1 patient also having an ASD], the presentation of a unicuspid aortic valve [2 patients 3%)], and patent foramen ovale [1 patient, (1%)]. Concomitant hyper-trabeculation of the left ventricle was observed in two patients (3%), whereas one patient presented significant left ventricular outflow tract obstruction due to the systolic anterior motion of an elongated AML without concomitant left ventricular hypertrophy. Finally, one patient was admitted to the emergency room because of complete atrioventricular block, with PML hypoplasia being a concomitant finding.

In the current literature, the management of patients with PML hypoplasia largely relied on patients’ clinical presentation. Patients with symptomatic severe MR underwent mitral valve replacement in isolation or in association with other interventions such as aortic valve replacement and ASD (Table 2). Mitral valve repair, including ring annuloplasty and chordal transfer from the posterior annulus to the anterior leaflet was performed in one patient [10].

The etiology of PML hypoplasia is not fully understood. The presence of familial cases [4], and the association with other congenital heart disease such as a bicuspid aortic valve [15,17,19,20], (Case 1 of our series) and ASD supports the hypothesis of a genetic contribution to the disease. The disruption in signaling transcription patterns involved in the development of the endothelia-to-mesenchymal transition might be potentially responsible for PML hypoplasia and other associated disorders [24].

There is no explication for the heterogenous clinical presentation of PML hypoplasia. In one of the larger existing monocentric case series [20], age was directly related to the severity of MR, supporting the hypothesis of disease progression from minor to severe MR over time. This evolution can be favored by the ongoing atrial and annular dilatation induced by MR itself, and/or by local hemodynamic conditions, which can portend PML fibrosis and retraction.

Our series of clinical cases is in line with the current findings in the literature, showing that PML hypoplasia can be a cause of symptomatic severe MR in adult patients, including the elderly.

TTE and TOE are fundamental for disclosing the etiology of MR in patients with PML and are useful in guiding the management of the disease. In young, symptomatic patients with severe MR, surgical mitral valve replacement seems to be the best therapeutic option. Nevertheless, one case of mitral valve repair has also been described [10].

In the elderly, the overall surgical risk should be evaluated, and less invasive strategies should be discussed. The anatomy of the hypoplastic PML generally contraindicates mitral TEER. Percutaneous mitral valve implantation can be proposed to selected patients with the appropriate anatomy after preliminary screening [1].

The presence of concomitant valvular or congenital heart disease should be systematically investigated in patients with PML hypoplasia, considering the frequent association of these diseases in the literature.

## 4. Conclusions

PML hypoplasia is a rare cause of primary MR in adults. However, the true prevalence of the disease is probably underdiagnosed because of asymptomatic patients.

Our case series and the analysis of the literature show that PML hypoplasia can remain asymptomatic, portending progressive MR and leading to the development of severe MR and symptoms only later in life.

Cardiologists should analyze the anatomy of the mitral valve carefully during routine TTE to identify PML hypoplasia, detect concomitant congenital cardiac disease, and provide the appropriate follow-up. In symptomatic patients, mitral valve surgery is probably the most useful approach. In the elderly, the management of the disease is more challenging, requiring heart team discussion and a multidisciplinary approach.

## Figures and Tables

**Table 1:** Main echocardiographic data.

	Case 1	Case 2	Case 3
LVEDV, mL	241	92	105
LVESV, mL	84	33	29
LVEF, %	65	64	70
LVESD, mm	43	23	
LAVi, mL/m²	50	77	68
ERO, cm²	0.9 cm²	0.42 cm²	0.51
RV, mL	95	85	72

ERO, effective regurgitant orifice; LAVi, indexed left atrial volume; LVEDV, left ventricular end-diastolic volume; LVEF, left ventricular ejection fraction; LVESD, left ventricular end-systolic diameter; LVESV, left ventricular end-systolic volume; and RV, regurgitant volume.

**Table 2:** Main publications on posterior mitral leaflet hypoplasia in adults.

AuthorsJournal DOI Number	N° of Cases	Age	Gender	Presentation Mitral Regurgitation Entity; Concomitant Heart Disease Disease/Symptoms at Presentation	Management
Bär, H. et al. (2009) [3]	3	Case 1: 62 yo	Woman	(1) Mild-to-moderate MR/Asymptomatic	Clinical follow-up
		Case 2: 62 yo	Man	(2) No MR (previous aortic valve surgery)/Asymptomatic	
		Case 3: 72 yo	Woman	(3) Mild MR/Mild AR (tricuspid valve)/Asymptomatic	
Kanagala, P. et al. (2010) [4]	3 (familial)	Case 1: 18 yo	Woman	(1) Mild MR/Asymptomatic	Clinical follow-up
		Case 2: 17 yo	Woman	(2) Mild MR/Asymptomatic	
		Case 3: 4- yo	Woman	(3) Mild MR/Asymptomatic	
Heper, G. et al. (2010) [5]	1	54 yo	Woman	Mild MR;ASD *ostrium secundum* type/Asymptomatic	Clinical follow-up
Okzan, H. et al. (2014) [6]	1	62 yo	Male	Severe MR/Dyspnea	MVR
de Agustin, J.A. et al (2014) [7]	1	73 yo	Woman	Severe MR/Dyspnea	MVR
Bezgin, T. et al. (2014) [8]	1	45 yo	Woman	Mild MR/Obstructive SAM	Betablocker therapy for dynamic LVOT
Saura, D. et al. (2015) [9]	1	51 yo	Male	Severe MR/Acute pulmonary oedema	MVR
Yazdan-Ashoori, P. et al. (2015) [10]	1	76 yo	Male	Severe MR/Acute pulmonary oedema	Mitral valve repair
Joshi, V. et al. (2014) [11]	1	66 yo	W	Severe MR/Dyspnea	MVR
Shah, J. et al. (2016) [12]	1	22 yo	Man	No MR/Asymptomatic	Clinical follow-up
Fazlinezhad, A. et al. (2017) [13]	1	24 yo	Man	Mild MR/Asymptomatic	Clinical follow-up
Bacich, D. et al. (2017) [14]	1	69 yo	Woman	Severe MR/Dyspnea	MVR
Parato, V.M. et al. (2018) [15]	2	Case 1 35 yo	Man	(1) Severe MR, LVNC, BAV/Dyspnea	(1) MVR
		Case 2 : 21 yo	Man	(2) Trace MR/Asymptomatic	(2) Clinical follow-up
Arasaratnam, K. et al. (2020) [16]	1	65	Woman	Severe MR/Dyspnea	MVR
Kadlečková, A. et al. (2021) [17]	2	Case 1: 59 yo Case 2: 48 yo	(1) Men	(1) Mild MR/Asymptomatic	(1) MVR, AVR
			(2) Man	(2) Severe MR; BAV; PFO, LVNC; previous ductus arteriosus surgery/AF	
Grandez, C. et al. (2022) [18]	1	22 yo	Man	No MR; Marphanoid habitus/ Asymptomatic	Clinical follow-up
Emami, E. et al. (2024) [19]	1	72 yo	Man	Severe MR, BAV already treated by AVR	Not indicated
Karaduman, A. et al. (2025) [20]	44	Median age: 31 yo (23-44.5)	22 men 22 women	14 moderate MR 11 severe MR 22 symptomatic (dyspnea) Concomitant disease: 13 BAV 2 unicuspid unicommissural aortic valve 7 ASD *ostium secundum* type 1 ASD *ostrium primum* type	7 MVR 14 MVR + other surgery 23 clinical follow-up
Antit, S. et al. (2025) [21]	1	30 yo	Man	No MR/Complete AV block	Clinical follow-up

ASD, atrial septal defect; AV, atrioventricular; AVR, aortic valve replacement; BAV, bicuspid aortic valve; LVNC, left ventricular non compaction; MR, mitral regurgitation; MVR, mitral valve replacement, PFO, patent foramen ovale; SAM, systolic anterior motion of the anterior mitral leaflet; and yo, year-old.

## Data Availability

No new data were created or analyzed in this study.

## Abbreviations

The following abbreviations are used in this manuscript:

AR	Aortic regurgitation
ASD	Anterior septal defect
BAV	Bicuspid aortic valve
ECG	Electrocardiogram
ERO	Effective regurgitant orifice
LAVi	Indexed left atrial volume
LVEDV	left ventricular end-diastolic volume
LVEF	left ventricular ejection fraction
LVESD	left ventricular end-systolic diameter
LVESV	left ventricular end-systolic volume
LVNC	Left ventricular non-compaction
MR	Mitral regurgitation
PML	Posterior mitral leaflet
RV	Regurgitant volume
TAVR	Transcatheter aortic valve replacement
TEER	Transcutaneous edge-to-edge repair
TEE	Transesophageal echocardiography
TTE	Transthoracic echocardiography
VHD	Valvular heart disease

## References

[1] Iung B., Delgado V., Rosenhek R., Price S., Prendergast B., Wendler O., De Bonis M., Tribouilloy C., Evangelista A., Bogachev-Prokophiev A. (2019). Contemporary Presentation and Management of Valvular Heart Disease: The EURObservational Research Programme Valvular Heart Disease II Survey. Circulation.

[2] Vahanian A., Beyersdorf F., Praz F., Milojevic M., Baldus S., Bauersachs J., Capodanno D., Conradi L., De Bonis M., De Paulis R. (2021). 2021 ESC/EACTS Guidelines for the management of valvular heart disease. Eur. Heart J..

[3] Bär H., Siegmund A., Wolf D., Hardt S., Katus H.A., Mereles D. (2009). Prevalence of asymptomatic mitral valve malformations. Clin. Res. Cardiol..

[4] Kanagala P., Baker S., Green L., Houghton A.R. (2010). Functionally uni-leaflet mitral valves in a family: A case series. Eur Heart J.-Cardiovasc. Imaging.

[5] Heper G., Yetkin E., Senen K. (2010). Absence of Posterior Mitral Leaflet With Secundum Atrial Septal Defect. Ann. Thorac. Surg..

[6] Ozkan H., Tiryakioglu O., Cetinkaya A.S., Uyanik E.C., Bozat T. (2014). Agenesis of the Mitral Posterior Leaflet in Elderly. Ann. Thorac. Surg..

[7] de Agustin J.A., de Diego J.J.G., Garcia-Fernandez M.A., Rodrigo J.L., Marcos-Alberca P., Almeria C., Nuñez-Gil I.J., Luaces M., Mahia P., Macaya C. (2014). Severe hypoplasia of the posterior mitral leaflet: A rare cause of congenital mitral regurgitation assessed by three-dimensional transesophageal echocardiography. Int. J. Cardiol..

[8] Bezgin T. (2014). Mitral valve with a single leaflet. Turk Kardiyol. Dernegi Arsivi-Archives Turk. Soc. Cardiol..

[9] Saura D., Oliva M.J., Sanchez-Galian M.J., Gonzalez J., Caballero L., Mateo-Martinez A., de la Morena G. (2015). Real-time three-dimensional transesophageal echocardiographic evaluation of the association of bicuspid aortic valve and mitral posterior leaflet hypoplasia. Int. J. Cardiol..

[10] Yazdan-Ashoori P., Rohani A., Mulji A.S., Van Spall H.G.C. (2015). Hypoplasia of the posterior mitral valve leaflet detected in late adulthood. Eur. Heart J..

[11] Joshi V., Laurie K., Skoyles J., Richens D. (2014). Severe Mitral Regurgitation Secondary to Atresia of the Posterior Mitral Valve Leaflet in the Adult: Is Repair Always Best Practice?. Thorac. Cardiovasc. Surg. Rep..

[12] Shah J., Jain T., Shah S., Mawri S., Ananthasubramaniam K. (2016). Rare Case of Unileaflet Mitral Valve. J. Cardiovasc. Ultrasound.

[13] Fazlinezhad A., Alvandi Azari M., Bigdellu L. (2016). Severe Hypoplasia of Posterior Mitral Valve Leaflet Presented with Atypical Chest Pain: A Case Report. Razavi Int. J. Med..

[14] Bacich D., Braggion G., Faggian G. (2017). Hypoplasia of the posterior mitral leaflet: A rare cause of mitral regurgitation in adulthood. Echocardiography.

[15] Parato V., Masia S. (2018). Hypoplasia or Absence of Posterior Leaflet: A Rare Congenital Anomaly of The Mitral Valve in Adulthood—Case Series. J. Cardiovasc. Echogr..

[16] Arasaratnam K., Tomlinson S., Dahiya A., Lo A., Jalali H., Prasad S.B. (2020). Surgical Repair of a Unileaflet Mitral Valve: A Rare Congenital Abnormality and a Novel Surgical Approach. CASE.

[17] Kadlečková A., Ioniţă O.R., Weichet J., Kačer P., Línková H. (2021). Hypoplasia of the posterior mitral leafl et—Are we familiar with it?. Cor Vasa.

[18] Grandez C., Skenderi S. (2023). Unileaflet Mitral Valve in Patient With Marfanoid Habitus. CASE.

[19] Emami E., Shemshadi S., Farrashi M. (2024). Aplastic Posterior Mitral Leaflet with an Unusual Clinical Course. Res. Cardiovasc. Med..

[20] Karaduman A. (2025). An Echocardiographic Study of a Rare Cause of Mitral Regurgitation: Hypoplastic Posterior Mitral Valve Leaflet. Anatol. J. Cardiol..

[21] Antit S., Soumer K., Abdelhedi M., Zidi O., Zakhama L. (2025). Incidental Findings of Congenital Unileaflet Mitral Valves in Young Patient Presenting Complete Atrioventricular Block. Pediatr. Cardiol..

[22] Remenyi B., Gentles T. (2012). Congenital mitral valve lesions: Correlation between morphology and imaging. Ann. Pediatr. Cardiol..

[23] Talner N.S., Stern A.M., Sloan H.E. (1961). Congenital Mitral Insufficiency. Circulation.

[24] Oosthoek P.W., Wenink A.C., Wisse L.J., Gittenberger-de Groot A.C. (1998). Development of the papillary muscles of the mitral valve: Morphogenetic background of parachute-like asymmetric mitral valves and other mitral valve anomalies. J. Thorac. Cardiovasc. Surg..

